# Methods for a Nonuniform Bose Gas

**DOI:** 10.6028/jres.101.046

**Published:** 1996

**Authors:** Kerson Huang, Paolo Tommasini

**Affiliations:** Center for Theoretical Physics, Laboratory for Nuclear Science, and Department of Physics, Massachusetts Institute of Technology, Cambridge, MA 02139; Institute for Theoretical Atomic and Molecular Physics, Harvard-Smithsonian Center for Astrophysics, Cambridge, MA 02138

**Keywords:** Bogoliubov transformation, Bose-Einstein condensation, Bose gas, Gaussian wave function, hard-sphere interaction, negative scattering length, pseudopotential, self-consistent field, variational method

## Abstract

We review mathematical methods for the treatment of a system of Bose particles with nonuniform density. The use of the pseudopotential is explained, especially with respect to negative scattering lengths. It is emphasized that the delta-function potential produces no scattering in three dimensions, and should not be used in the Bogoliubov self-consistent field method, which is variational in nature. A common misuse of the Bogoliubov method at finite temperatures is pointed out. A Gaussian variational method is proposed.

## 1. Introduction

The observation of Bose-Einstein condensation in atomic traps [1]–[3] has renewed theoretical interest in studying a system of Bose particles with a nonuniform density. The experiments involve atoms of mass *m* condensed in a harmonic trap characterized by frequency *ω*. The relevant parameters, as discussed more fully in Refs. [4] and [5], have the following orders of magnitude:

$$\begin{array}{l} a\sim 10^{-6}\mathrm{cm},\\ \lambda ={\left(2\pi \hbar^{2}/mkT\right)}^{1/2}\sim 10^{-4}\mathrm{cm},\\ L=(\hbar /m\omega {)}^{1/2}\sim 10^{-4}\mathrm{cm},\\ na^{3}\sim 10^{-8}, \end{array}$$ (1)

where *a* is the *s*-wave scattering length, *λ* the thermal wavelength, *L* the size of the system, and *n* is the average particle density. Thus, *na*^3^ << 1, *a*/*L* << 1, *a*/*l* << 1, and we can treat the system as a low-temperature weakly-interacting dilute gas using well-known methods [6].

In this paper, we review the following theoretical methods, and point out some common errors and misconceptions:

- The pseudopotential method: One replaces the potential by a *δ*-function, and, with the help of a Bogoliubov transformation, obtains a weak-coupling expansion. This method seems adequate to describe current experiments.
- The self-consistent field method: This is variational, and capable of treating strongly interacting cases in principle. It reduces to the pseudopotential method in appropriate limits; but one should not use a *δ*-function potential in a variational calculation, because such a potential in three spatial dimensions has no effect, if treated exactly.
- The Gaussian variational method: One uses a Gaussian trial wave function, and obtains results similar to the self-consistent field method. It has the advantage of easily adaptable to the description of time-dependent phenomena.

All three methods are closely related to one another. Although we will not use Feynman graphs, it might be relevant to note that the Bogoliubov transformation corresponds to summing “one-loop” graphs. The self-consistent field method consists of readjusting parameters in the one-loop sum in a variational sense. The Gaussian method includes all one-loop contribution, plus parts of higher-loop contributions. Of these methods, the straightforward one-loop summation yields an expansion in *na*^3^. On the other hand, the accuracy of the other methods cannot be ascertained.

We do not attempt a comprehensive review, and the references cited are by no means complete. In a subject with such a long history, it is difficult to be aware of all relevant sources, and we apologize for any omissions [7].

## 2. Pseudopotential Method

In low-temperature calculations, one can replace the interatomic potential with a *δ*-function potential (4π*aℏ*^2^/*m*)*δ*(***r***), where *a* > 0 is the *s*-wave scattering length. Such a potential should be treated in low-order perturbation theory only, because the *δ*-function potential in three spatial dimensions has no effect if treated exactly [8].

Consider the Schrödinger equation

$$-\nabla^{2}\psi (r)+g\delta^{3}(r)\psi (r)=E\psi (r),$$ (2)

where *E* is 2*m*/*ℏ*^2^ times the energy. Taking the Fourier transform of both sides leads to

$$(E-k^{2})\phi (k)=g\psi (0),$$ (3)

where *ϕ*(***k***) is the Fourier transform of *ψ* (***r***). This must hold for all ***k***, including *k*^2^ = *E*. Therefore *ψ*(0) = 0. This condition is automatically satisfied for all partial waves except *s*-waves. For *s*-waves, this seems to require that *ψ*(***r***) be discontinuous at ***r*** = 0; but because of the singular nature of the potential at ***r*** = 0, one should be careful. By treating the *δ*-function as the limit of a square well, one can verify that in three dimensions the *s*-waves are the same as those of a free particle except at ***r*** = 0, where they drop to zero discontinuously. This behavior, however, does not lead to any scattering, nor level shift.

As a more careful analysis [9] shows, the *δ*-function potential must be regarded as the first term in a pseudo-potential, which correctly reproduces all scattering phase shifts, and depends on these phase shifts as input parameters. For the simple case of a hard-sphere potential, for which there is only one parameter *a*, the hard-sphere diameter, the pseudopotential has the form

$$\begin{array}{l} V_{\mathrm{pseudo}}(r)=\\ \frac{4\pi a\hbar^{2}}{m}\left[\delta^{3}(r)\frac{\partial }{\partial r}r-\frac{a^{2}}{3}\delta^{3}(r)\nabla^{2}\frac{\partial }{\partial r}r+\cdots \right], \end{array}$$ (4)

The differential operator (∂/∂*r*)*r* removes spurious singularities in the wave function, which would otherwise make the ground-state energy diverge. In low-order perturbation theory, one can replace this operator by a simple subtraction procedure for the energy.

Using the first term in the pseudopotential, and making a Bogoliubov transformation, one can calculate exactly the first few terms of an expansion for the ground-state energy per particle of a uniform hard-sphere Bose gas [10, 11]:

$$\begin{array}{l} E_{0}=\frac{2\pi na\hbar }{m}[1+\frac{128}{15}{\left(\frac{na^{3}}{\pi }\right)}^{1/2}\\ +8\left(\frac{4\pi }{3}-\sqrt{3}\right)na^{3}\ln(na^{3})+O(na^{3})]. \end{array}$$ (5)

This shows that the energy is not analytic at *a* = 0, and therefore cannot be continued to negative values of *a*.

Fetter [12] has given a systematic treatment of the nonuniform case based on the Bogoliubov transformation. For application to the atomic-trap experiments, one can also adapt the results of the uniform case using a Thomas-Fermi approximation [5].

What happens when the scattering length is negative? In this case, the two-body scattering is dominated by the attractive part of the potential. The attraction may or may not be strong enough to produce a two-particle bound state; but it will lead to an *N*-body bound state, for a sufficiently large *N*. The many-body problem is very different from that of the repulsive case, for it depends on the details of the potential. Consider two potentials with the same negative scattering length, one everywhere attractive, and the other having a repulsive core. The many-body system with the purely attractive potential will collapse spatially, whereas the one with hard core will yield extensive energies.

When the scattering length is negative, we must therefore use an actual potential, for example, one with a hard core plus an attractive tail. We can replace the hard-core part by a pseudopotential, and do the Bogoliubov transformation. In this manner, it has been shown [6] [13] that a dilute Bose system with such a potential exhibits a first-order phase transition separating a gas phase from a liquid phase. The transition line terminates in a critical point. The Bose-Einstein condensation occurs in the liquid phase, and divides it into liquid I and liquid II. In short, one reproduces qualitatively the phase diagram of ^4^He. Since the calculation is perturbative, one can follow the collapse of the phase diagram to that of the ideal Bose gas when the potential is turned off.

The results described above pertains to an infinite Bose system in the thermodynamic limit, and may be modified for a finite system. The authors of Ref. [2] observed Bose condensation in an atomic trap filled with in ^7^Li, which has a negative scattering length. In this case, the smallness of the system may have rendered the first-order transition indistinct. It is also possible that the observed phenomenon is metastable.

## 3. Variational Methods and the Energy Gap

A variational approach to the ground-state energy of a Bose gas was introduced by Girardeau and Arnowitt [14], who use (wisely) an actual potential instead of the *δ*-function. Their results are for a uniform gas, but otherwise very similar to what we obtain later in the self-consistent field method. The excitation spectrum in this approach, however, contains an energy gap, contrary to general expectations [15], and to the perturbation-theory results in the hard-sphere case [10].

A variational method is designed to yield the best ground-state energy, and may not yield the excitation spectrum accurately. In this instance, we know that the gap should have been filled with phonons, which can be described using general principles. We may therefore view the variational principle as a way to obtain an approximate ground-state wave function, and build the phonon states upon this ground state.

Feynman [16] argues that the low-lying excited states of a Bose system are purely phonon states, owing to the statistics, and suggests the following one-phonon wave function of momentum ***k***:

$$\Psi_{k}(r_{1,\ldots ,}r_{N})=\sum_{j=1}^{N}e^{ik\cdot r_{j}}\Psi_{0}(r_{1,\ldots ,}r_{N}),$$ (6)

where *Ψ*_0_ is the ground-state wave function. The excitation energy is

$$E_{k}=\frac{\hbar^{2}k^{2}}{2mS_{k}},$$ (7)

where *S_k_* is the liquid structure factor

$$S_{k}=\frac{1}{n}\langle \Psi_{0}\left|{\xi_{k}}^{†}\xi_{k}\right|\Psi_{0}\rangle ,$$ (8)

where

$$\xi_{k}=\Omega^{-1/2}\sum_{p}a_{p+k}^{†}a_{p},$$ (9)

with *a_k_* the annihilation operator of a free particle of momentum *ℏ****k***, and *Ω* the volume. From general principles [17,18], we expect

$$S_{k}\underset{k\to 0}{\to }\frac{\hbar k}{2mc},$$ (10)

where *c* is the sound velocity as computed from the compressibility of the ground state. This leads to the phonon spectrum

$$E_{k}=\hbar ck.$$ (11)

Feynman’s argument can be sharpened by recasting it as the statement that the longitudinal sum-rules are saturated by the phonon states [17] [18]. All the equations above are verified in the dilute hard-sphere Bose gas [10] [18].

Other types of states that can be built upon *Ψ*_0_ are those describing superfluid flow [10] [18]:

$$\Psi_{\text{superflow}}(r_{1,\ldots ,}r_{N})=\prod_{j}^{N}e^{i\alpha (r_{j})}\Psi_{0}(r_{1,\ldots ,}r_{N}),$$ (12)

where *α* (***r***) is the superfluid phase, which is related to the superfluid velocity ***v****_s_* through

$$v_{s}(r)=\frac{\hbar }{m}\nabla \alpha (r).$$ (13)

With this wave function one can describe quantized vortices.

## 4. Self-Consistent Field Method: Ground State

The self-consistent field method was first used by Bogoliubov in his treatment of superconductivity, and is therefore also known as the “Bogoliubov method.” We shall follow a concise formulation due to De Gennes [19]. A recent review of similar methods is given by Griffin [20].

We use a quantized-field representation with field operator *Ψ* (***x***), and canonical conjugate *iΨ*^†^(***x***):

$$[\Psi (x),\Psi^{†}(y)]=\delta^{3}(x-y).$$ (14)

The Hamiltonian is

$$\begin{array}{c} H=\int d^{3}x\Psi {\left(x\right)}^{†}h(x)\Psi (x)\\ +\frac{1}{2}\int d^{3}xd^{3}y\Psi {\left(y\right)}^{†}\Psi {\left(x\right)}^{†}V(x,y)\Psi (x)\Psi (y), \end{array}$$ (15)

where

$$h(x)=-\frac{\hbar^{2}}{2m}\nabla^{2}+V_{\mathrm{ext}}(x)-\mu ,$$ (16)

where *V*_ext_(***x***) is the external potential, and *μ* is the chemical potential. We displace the field operator by its ground-state expectation:

Ψ(x)=ψ(x)+ϕ(x), (17)

where *ψ* (***x***) is the displaced field operator, and

ϕ(x)=〈ψ(x)〉. (18)

The function *ϕ* (***x***) describes a nonuniform condensate, and *ψ* (***x***) annihilates particles not in the condensate.

Variational trial states are taken to be the eigenstates of an effective Hamiltonian that is quadratic in the field operators, chosen to be a mean-field version of the actual Hamiltonian. There are two variational functions *ρ* (***x***,***y***) and *Δ* (***x***,***y***), which will turn out to be *ρ* (***x***,***y***) = 〈*ψ*^†^(***x***) *ψ* (***y***)〉, and *Δ* (***x***,***y***) = 〈*ψ* (***x***)*ψ* (***y***)〉. That is, *ρ* and *Δ* are, respectively, the direct and off-diagonal density correlation functions of the uncondensed particles.

The effective Hamiltonian is chosen to be

$$H_{\mathrm{eff}}=H^{\left(0\right)}+H^{\left(1\right)}+H^{(1)†}+H^{\left(2\right)},$$ (19)

where *H*^(0)^ is independent of *ψ*, and *H*^(1)^ and *H*^(2)^ terms are respectively linear and quadratic in *ψ*:

$$\begin{array}{c} H^{\left(0\right)}=\int d^{3}x\phi^{*}(x)h(x)\phi (x)\\ +\frac{1}{2}\int d^{3}xd^{3}y\phi^{*}(y)\phi^{*}(x)V(x,y)\phi (x)\phi (y), \end{array}$$ (20)

$$\begin{array}{c} H^{\left(1\right)}=\int d^{3}x\psi^{†}(x)h(x)\phi (x)\\ +\int d^{3}xd^{3}yV(x,y)\psi^{†}(x)[\phi (y)\rho (y,x)+\phi (x)\rho (y,y)\\ +\phi^{*}(y)D(x,y)], \end{array}$$ (21)

$$\begin{array}{c} H^{\left(2\right)}=\int d^{3}x\psi^{†}(x)h(x)\psi (x)\\ +\int d^{3}xd^{3}yV(x,y)\;[\psi^{†}(y)\psi (x)R(x,y)\\ +\psi^{†}(x)\psi (x)R(y,y)]\\ +\frac{1}{2}\int d^{3}xd^{3}yV(x,y)\;[\psi^{†}(x)\psi^{†}(y)D(x,y)\\ +\psi (x)\psi (y)D^{*}(x,y)], \end{array}$$ (22)

where

$$\begin{array}{l} R(x,y)\equiv \rho (x,y)+\phi^{*}(x)\phi (y),\\ D(x,y)\equiv \Delta (x,y)+\phi (x)\phi (y). \end{array}$$ (23)

For arbitrary *ρ* and *Δ*, we can diagonalize the effective Hamiltonian through a generalized Bogoliubov transformation:

$$\psi (x)=\sum_{n}\left[u_{n}(x)\eta_{n}-v_{n}^{*}(x)\eta_{n}^{†}\right].$$ (24)

where η*_n_* and $\eta_{n}^{†}$ are annihilation and creation operators satisfying

$$[\eta_{n},\eta_{m}^{†}]=\delta_{nm}.$$ (25)

To preserve the canonical commutators for *ψ* (***x***), we require

$$\sum_{n}\left[u_{n}(x)u_{n}^{*}(y)-v_{n}^{*}(x)v_{n}(y)]=\delta^{3}(x-y\right).$$ (26)

We now require *H*_eff_ to have the form

$$H_{\mathrm{eff}}=E_{0}+\sum_{n}\epsilon_{n}\eta_{n}^{†}\eta_{n},$$ (27)

or, equivalently,

$$\begin{array}{l} {}[H_{\mathrm{eff}},\eta_{n}]=-\epsilon_{n}\eta_{n},\\ {}[H_{\mathrm{eff}},\eta_{n}^{†}]=\epsilon_{n}\eta_{n}^{†}. \end{array}$$ (28)

These conditions determine the functions *v_n_*, *u_n_* and *ϕ*. The condition for *ϕ* comes from the requirement that in *H*_eff_ the terms linear in *ψ*(***x***) vanish. The resulting equations are

$$\begin{array}{c} h(x)\phi (x)+\int d^{3}yV(x,y)\;[\phi (y)\rho (y,x)+\phi (x)\rho (y,y)\\ +\phi^{*}(y)\Delta (x,y)+{\left|\phi (y)\right|}^{2}\phi (x)]=0, \end{array}$$ (29)

$$\begin{array}{c} \epsilon_{n}u_{n}(x)=h(x)u_{n}(x)+\int d^{3}yV(x,y)\;[u_{n}(x)R(y,y)\\ +\;u_{n}(y)R(y,x)-v_{n}(y)D(x,y)], \end{array}$$ (30)

$$\begin{array}{c} -\epsilon_{n}v_{n}(x)=h(x)v_{n}(x)+\int d^{3}yV(x,y)\;[v_{n}(x)R(y,y)\\ +v_{n}(y)R(y,x)-u_{n}(y)D^{*}(x,y)]. \end{array}$$ (31)

To determine *ρ* and *Δ*, let |*Φ*〉 be the lowest eigenstate of *H*_eff_:

$$H_{\mathrm{eff}}|\Phi \rangle =E_{0}|\Phi \rangle .$$ (32)

We require that the ground-state energy be at a minimum with respect to variations in *ρ* and *Δ*:

δ〈H〉=0, (33)

where the brackets denote expectation value with respect to |*Φ*〉. In calculating the expectation value above, we use Wick’s theorem:

$$\begin{array}{c} \langle \psi^{†}\left(x\right)\psi^{†}\left(y\right)\psi (x)\psi \left(y\right)\rangle =\langle \psi^{†}\left(x\right)\psi \left(y\right)\rangle \langle \psi^{†}\left(y\right)\psi \left(x\right)\rangle \\ +\langle \psi^{†}(x)\psi (x)\rangle \langle \psi^{†}\left(y\right)\psi \left(y\right)\rangle +\langle \psi^{†}\left(x\right)\psi^{†}\left(y\right)\rangle \langle \psi \left(x\right)\psi \left(y\right)\rangle . \end{array}$$ (34)

The calculation is facilitated by noting that δ 〈*H*_eff_〉 = 0, since *Φ* is an eigenstate. Thus we can use the equivalent condition

$$\delta \langle H-H_{\mathrm{eff}}\rangle =0.$$ (35)

The results are as follows:

$$\begin{array}{l} \rho (x,y)=\langle \psi^{†}\left(x\right)\psi \left(y\right)\rangle =\sum_{n}v_{n}(x)v_{n}^{*}(y),\\ \Delta (x,y)=\langle \psi \left(x\right)\psi \left(y\right)\rangle =-\sum_{n}u_{n}(x)v_{n}^{*}(y). \end{array}$$ (36)

When these are substituted into Eqs. (29), (30), and (31), we have a system of coupled nonlinear integro-differential equations.

## 5. The Uniform Case

We put *V*_ext_ = 0, assume *V*(***x***,***y***) = *V*(***x*** − ***y***), and seek solutions with uniform density by putting

$$\begin{array}{l} \phi (r)=\phi_{0},\\ u_{k}(r)=\Omega^{-1/2}e^{ik\cdot r}x_{k},\\ v_{k}(r)=\Omega^{-1/2}e^{ik\cdot r}y_{k}, \end{array}$$ (37)

where *x_k_*, *y_k_* and *ϕ*_0_ can be taken to be real. The condition [Eq. (26)] becomes $x_{k}^{2}-y_{k}^{2}=1$, which can be satisfied by putting

$$\begin{array}{l} x_{k}=\cosh\sigma_{k},\\ y_{k}=\sinh\sigma_{k}. \end{array}$$ (38)

This parametrization simplifies the calculations. For example, the liquid-structure factor of the ground state can be represented in the form

$$\begin{array}{c} S_{k}=1+\frac{n_{0}}{n}(\cosh2\sigma_{k}-\sinh2\sigma_{k}-1)\\ +\frac{1}{4n\Omega }\sum_{p}[\sinh2\sigma_{p}\sinh2\sigma_{\left|k+p\right|}\\ +\sinh^{2}\sigma_{p}\sinh^{2}\sigma_{\left|p+k\right|}], \end{array}$$ (39)

where *n*_0_ is the condensate density given through

$$n=n_{0}+\Omega^{-1}\sum_{p}\sinh^{2}\sigma_{p}.$$ (40)

The Bogoliubov result is recovered by neglecting the ***p*** sums, which is equivalent to putting *n*_0_ ≈ *n*:

$$S_{k}={\left[\frac{\epsilon_{k}}{\epsilon_{k}+2nV_{k}}\right]}^{1/2},$$ (41)

where **ϵ***_k_* = *ℏ*^2^*k*^2^/2*m*, and *V_k_* is the Fourier transform of the interaction potential.

## 6. Reduction to *δ*-Function Potential

The equations in the last section are rather complex, and difficult to solve even numerically. To gain some insight, we reduce them to a simpler form by using a *δ*-function potential. We shall show that this potential has no effect if self-consistency is enforced, at least in the case of a uniform system. We put

$$V(x,y)=g\delta^{3}(x-y),$$ (42)

where

$$g=4\pi \hbar^{2}a/m.$$ (43)

The functions *ρ* and *Δ* now depend only on ***x***:

$$\begin{array}{l} \rho \left(x\right)=\langle \psi^{†}\left(x\right)\psi \left(x\right)\rangle =\sum_{n}V_{n}\left(x\right)V_{n}^{*}\left(x\right)\\ \Delta \left(x\right)=\langle \psi \left(x\right)\psi \left(x\right)\rangle =-\sum_{n}u_{n}\left(x\right)v_{n}^{*}\left(x\right). \end{array}$$ (44)

Note that *ρ* is the depletion of the condensate due to interactions. Equations (29), (30), and (31) reduce to

$$\left[h+2g\rho +g{\left|\phi \right|}^{2}\right]\phi +g\Delta \phi^{*}=0,$$ (45)

$$\begin{array}{l} \left(h+2g\rho +2g\phi^{*}\phi \right)u_{n}-g\left(\Delta +\phi^{2}\right)v_{n}=\epsilon u_{n},\\ \left(h+2g\rho +2g\phi^{*}\phi \right)v_{n}-g\left(\Delta^{*}+\phi^{*2}\right)u_{n}=-\epsilon v_{n}, \end{array}$$ (46)

where we have suppressed the ***x*** dependence of the functions. The dilute uniform hard-sphere gas is recovered by setting *ρ* = *Δ* = 0, for they give higher-order contributions.

For *ρ* = *Δ* = 0, Eq. (45) is the “nonlinear Schrödinger equation” [21] [22], which has been widely used in numerical studies of the condensate and its excitations [23]. One should be aware, however, that *ρ* and *Δ* may be important, especially for the excitations.

In the uniform case, with *V*_ext_ = 0, we obtain, in the notation of the last section,

$$\tanh\;2\sigma_{k}=\frac{g\left(n-\rho +\Delta \right)}{\epsilon_{k}+g\left(n-\rho -\Delta \right)},$$ (47)

where ***ϵ****_k_* = *ℏ*^2^*k*^2^/2*m*, and the chemical potential *μ* has been eliminated in terms of the density *n*. The self-consistency conditions [Eq. (44)] then lead to

$$\begin{array}{l} \rho =\frac{1}{4\pi^{2}}\int_{0}^{\Lambda }dkk^{2}\{f_{k}[\epsilon_{k}+g(n-\rho -\Delta )]-1\},\\ \Delta =-\frac{g}{4\pi^{2}}\int_{0}^{\Lambda }dkk^{2}f_{k}(n-\rho -\Delta ), \end{array}$$ (48)

where

$$f_{k}={\left[\epsilon_{k}^{2}+2g\epsilon_{k}\left(n-\rho -\Delta \right)-4g^{2}\left(n-\rho \right)\Delta \right]}^{-1/2}.$$ (49)

The integrals are cut off at *Λ*, because otherwise *Δ* would be divergent. Solving for *Δ* from the second equation, we obtain in the limit *Λ* → ∞

Δ−ρ+n=0. (50)

The first equation then gives, in the limit *Λ* → ∞,

ρ=0. (51)

That is, there is no depletion of the condensate. It is then straightforward to show that the system is an ideal Bose gas. This result is reassuring; it shows that the method is able to verify that the *δ*-function potential has no effect.

## 7. Self-Consistent Field Method: Finite Temperatures

To extend the variational approach to finite temperatures, we can use the eigenstates of *H*_eff_ to calculate the partition function, and then minimize the free energy

F=〈H〉−TS, (52)

where the brackets now denote thermal average with respect to *H*_eff_, and *S* is the entropy. De Gennes [19] argues that the quantity

$$F_{\mathrm{eff}}=\langle H_{\mathrm{eff}}\rangle -TS$$ (53)

is stationary, and implements the variational principle by minimizing *F* − *F*_eff_. But the assertion is correct only if *S* were defined with respect to the eigenvalue spectrum of *H*_eff_. In fact, as we explain below, *S* is defined with respect to the energy levels 〈*α*|*H*|*α*〉, where |*α*〉 are the eigenstates of *H*_eff_. The finite-temperature results in Ref. [19] are therefore incorrect.

A proper variational principle for the free energy [24] is based on the inequality

$$\sum_{\alpha }\langle \alpha \left|e^{-\beta H}\right|\alpha \rangle \geq \sum_{\alpha }e^{-\beta \langle \alpha \left|H\right|\alpha \rangle }.$$ (54)

This shows that, to calculate the trial partition function, we must use the energy spectrum

$$W_{\alpha }=\langle \alpha \left|H\right|\alpha \rangle .$$ (55)

In contrast, the other scheme leads to a free Bose gas with single-particle energy **ϵ***_n_*, the eigenvalues from Eqs. (30) and (31). Because of the simplicity, it is widely used in the literature; but it does not follow from a variational principle.

As an example of the difference between the two schemes, the calculation of the partition of a hard-sphere Bose gas in Ref. [25] corresponds to using Eq. (55), and yields the virial coefficients in the gas phase. On the other hand, a calculation based on the other scheme would give a free Bose gas above the transition temperature.

## 8. Gaussian Variational Method

We go to a representation in which the field operator *Ψ* (***x***) and its conjugate have the forms

$$\begin{array}{l} \Psi (x)=\frac{1}{\sqrt{2}}\left[\varphi (x)+\frac{\delta }{\delta \varphi (x)}\right],\\ i\Psi^{†}(x)=\frac{i}{\sqrt{2}}\left[\varphi (x)-\frac{\delta }{\delta \varphi (x)}\right], \end{array}$$ (56)

where *φ* (***x***) is a *c*-number function. The more familiar representation, in which *Ψ* is diagonal, would be awkward to use in a non-relativistic case, in which *Ψ* and *iΨ*^†^ are canoncial conjugates. (A similar situation happens in a relativistic fermion field obeying the Dirac equation.)

The state of the system is described by a wave functional *Ψ* [*φ*]. We use a Gaussian form for a trial wave functional:

$$\begin{array}{l} \Phi \left[\varphi \right]=C\exp\{-\frac{1}{4}\int d^{3}xd^{3}y[\varphi \left(x\right)\\ -\varphi_{0}\left(x\right)]G^{-1}\left(x,y\right)\left[\varphi \left(y\right)-\varphi_{0}\left(y\right)\right]\}, \end{array}$$ (57)

where *C* is a normalization constant and *φ*_0_ and *G*(***x***,***y***) are the variational parameters. Expectation values are given by functional integrals, for example

$$\langle H\rangle =\int \left(D\varphi \right)\Phi^{*}\left[\varphi \right]H\Phi \left[\varphi \right]$$ (58)

where *Φ* is normalized to unity. It is easy to show that

$$\begin{array}{c} \langle \Psi \left(x\right)\rangle =\varphi_{0}\left(x\right),\\ \langle \varphi \left(x\right)\varphi \left(y\right)\rangle =G\left(x,y\right)+\varphi_{0}\left(x\right)\varphi_{0}\left(y\right). \end{array}$$ (59)

The variational principle states that

$$\frac{\delta \langle H\rangle }{\delta G\left(x,y\right)}=0,\;\frac{\delta \langle H\rangle }{\delta \varphi_{0}\left(x\right)}=0.$$ (60)

The equation obtained for *G* and *φ*_0_ will not be given directly. To make contact with the self-consistent field method, we quote the results

$$\begin{array}{c} \rho \left(x,y\right)\equiv \langle \psi {\left(x\right)}^{†}\psi \left(y\right)\rangle \\ =\frac{1}{2}\left[\frac{1}{4}G^{-1}\left(x,y\right)+G\left(x,y\right)-\delta^{3}\left(x-y\right)\right],\\ \Delta \left(x,y\right)\equiv \langle \psi \left(x\right)\psi \left(y\right)\rangle \\ =-\frac{1}{2}\left[\frac{1}{4}G^{-1}\left(x,y\right)-G\left(x,y\right)\right]. \end{array}$$ (61)

For further analysis, it is convenient to introduce

$$\begin{array}{l} X\left(x,y\right)\equiv \frac{1}{2\sqrt{2}}\left[G^{-1/2}\left(x,y\right)+2G^{1/2}\left(x,y\right)\right].\\ Y\left(x,y\right)\equiv \frac{1}{2\sqrt{2}}\left[G^{-1/2}\left(x,y\right)-2G^{1/2}\left(x,y\right)\right]. \end{array}$$ (62)

it easy to show that *ρ* (***x***,***y***) = ∫ d^3^*zY*(***x***,***z***)*Y*(***z***,***y***), and *Δ* (***x***,***y***) = − ∫ d^3^*zX*(***x***,***z***)*Y*(***z***,***y***). Now expand *X* and *Y* in terms of a basis {*b_n_*(***x***)}:

$$\begin{array}{l} X\left(x,y\right)=\sum_{n}X_{n}b_{n}\left(x\right)b_{n}^{*}\left(y\right),\\ Y\left(x,y\right)=\sum_{n}Y_{n}b_{n}\left(x\right)b_{n}^{*}\left(y\right), \end{array}$$ (63)

where *X_n_* and *Y_n_* are real. We then find

$$\begin{array}{l} \rho \left(x,y\right)=\sum_{n}b_{n}\left(x\right)b_{n}^{*}\left(y\right)Y_{n}^{2},\\ \Delta \left(x,y\right)=-\sum_{n}b_{n}\left(x\right)b_{n}^{*}\left(y\right)X_{n}^{2}. \end{array}$$ (64)

Comparison with Eq. (36) leads to the identification

$$\begin{array}{l} u_{n}\left(x\right)=b_{n}\left(x\right)X_{n},\\ v_{n}\left(x\right)=b_{n}\left(x\right)Y_{n}. \end{array}$$ (65)

This shows the equivalence between this method and the self-consistent field method.

One of the advantages of the Gaussian method lies in the possibility of generalization to the “time-dependent Hartree-Fock” method [26,27], which has been used successfully in nuclear physics. One of us (P.T.) plans to discuss this in a separate publication.
